# Current Challenges and Opportunities in Non-native Chemical Production by Engineered Yeasts

**DOI:** 10.3389/fbioe.2020.594061

**Published:** 2020-12-14

**Authors:** Jiwon Kim, Phuong Hoang Nguyen Tran, Sun-Mi Lee

**Affiliations:** ^1^Clean Energy Research Center, Korea Institute of Science and Technology (KIST), Seoul, South Korea; ^2^Department of Biotechnology, Korea University, Seoul, South Korea; ^3^Division of Energy and Environment Technology, University of Science and Technology (UST), Daejeon, South Korea; ^4^Green School, Korea University, Seoul, South Korea

**Keywords:** non-native chemicals, *Saccharomyces cerevisiae*, *Yarrowia lipolytica*, yeast engineering, biorefinery

## Abstract

Yeasts are promising industrial hosts for sustainable production of fuels and chemicals. Apart from efficient bioethanol production, yeasts have recently demonstrated their potential for biodiesel production from renewable resources. The fuel-oriented product profiles of yeasts are now expanding to include non-native chemicals with the advances in synthetic biology. In this review, current challenges and opportunities in yeast engineering for sustainable production of non-native chemicals will be discussed, with a focus on the comparative evaluation of a bioethanol-producing *Saccharomyces cerevisiae* strain and a biodiesel-producing *Yarrowia lipolytica* strain. Synthetic pathways diverging from the distinctive cellular metabolism of these yeasts guide future directions for product-specific engineering strategies for the sustainable production of non-native chemicals on an industrial scale.

## Introduction

Microorganisms have gained significant attention as cell factories for the sustainable production of fuels and chemicals, providing great opportunities for a bio-based economy in the post-petroleum era. Of the microbial cell factories, yeast has long been used as a proven industrial producer of fuels and chemicals. The robustness in a wide range of environmental conditions, the ease of separation from the culture medium, and no phage contamination related issues make the yeast more attractive industrial cell factory than bacteria. With recent advances in synthetic biology, the product profiles of yeasts have been expanding to include those that have never been produced naturally, such as cannabinoids, tropine, and amorphadiene (Luo et al., 2019; Ping et al., 2019; Srinivasan and Smolke, 2019; Marsafari and Xu, 2020). Given that synthetic pathways for non-native products commonly involve multiple enzymes from plants and/or other higher eukaryotes, which often require post-translational modification and are difficult to be functionally expressed in prokaryotic systems, the development of yeast cell factories is drawing more attention with its advantages as a eukaryotic system.

The model yeast *Saccharomyces cerevisiae* is one of the most widely developed cell factories for the production of fuels and chemicals. Industrial production of ethanol, the native product of *S. cerevisiae*, is in operation worldwide to supply alcoholic beverages and biofuels (Walker and Stewart, 2016; Parapouli et al., 2020). Industrial-scale production of β-farnesene by engineered *S. cerevisiae* strains has also been reported (Gray et al., 2014; Wehrs et al., 2019). Of the non-model yeast, *Yarrowia lipolytica* has recently risen as another promising industrial cell factory with a superior capacity to accumulate lipids for biodiesel production (Ma et al., 2019). Currently, taking advantage of readily accessible genetic information and highly efficient genetic tools, an increasing number of products are being added to the product profiles of the model yeast *S. cerevisiae.* Besides being utilized for biodiesel production, *Y. lipolytica* is being transformed into a cell factory platform for the production of advanced biofuels and chemicals such as isoprenoids, polyketides, drug precursors, pigments, and organic acids (Miller and Alper, 2019).

**FIGURE 1:**
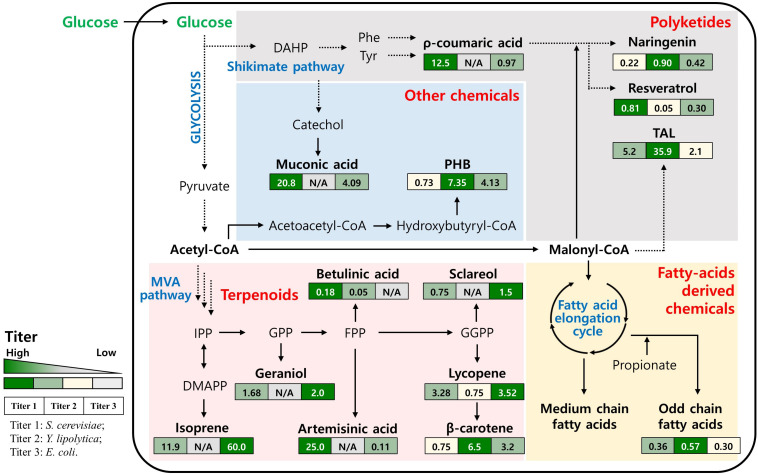
Simplified biosynthetic pathway and the main intermediates for production of non-native chemicals in yeasts. The values in the boxes represent the production titer obtained by engineered *S. cerevisiae*, *Y. lipolytica* and *E. coli* (from left to right). N/A, not available; DAHP, 3-Deoxy-D-arabinoheptulosonate 7-phosphate; Phe, Phenylalanine; Tyr, Tyrosine; TAL, Triacetic acid lactone; PHB, Polyhydroxybutyrate; IPP, Isopentenyl pyrophosphate; DMAPP, Dimethylallyl pyrophosphate; GPP, Geranyl pyrophosphate; FPP, Farnesyl pyrophosphate; GGPP, Geranylgeranyl diphosphate.

The successful use of *S. cerevisiae* for the industrial production of bioethanol and the superior lipid production performance of *Y. lipolytica* shows that these strains can provide an economically feasible method for carbohydrate-based-biofuels production (Qiao et al., 2017). As such, there is increasing hope that these strains could be used for the production of numerous non-native chemicals on an industrial scale. Their GRAS (generally recognized as safe) status, as well as their well-known genomic characteristics and widely available engineering tools, make these yeast strains attractive microbial cell factories for the production of numerous chemicals (Darvishi et al., 2017; Markham and Alper, 2018; Ekas et al., 2019; Favaro et al., 2019). Here, the recent advances in engineering *S. cerevisiae* and *Y. lipolytica* as yeast cell factories for the production of non-native chemicals will be reviewed. Non-native chemicals were classified into four large groups, (i) terpenoids, (ii) polyketides, (iii) fatty acid-derived chemicals, and (iv) others, based on the core intermediate used as a precursor, namely acetyl-CoA, malonyl-CoA, and DHAP, which are key intermediates and diverge into branched pathways in the cellular metabolism of yeasts, such as the mevalonate or shikimate pathways (Figure 1). Thus, engineering strategies to produce certain groups of chemicals could be shared and more easily expanded to transform yeasts into cell factories for specific non-native chemicals. The performance of engineered yeasts as producers of specific chemical groups was analyzed and compared to that of the intensively engineered bacterial host of *E. coli* to highlight the benefits of using engineered yeasts for the industrial production of non-native chemicals by maximizing their potential, which is supported by their distinctive cellular metabolism. Finally, future directions for choosing an optimal platform strain and engineering strategies will be discussed to advance yeast cell factories for the sustainable production of non-native chemicals on an industrial scale.

## Production of Non-Native Chemicals in Yeast Cell Factories

### Terpenoids

Terpenoids, also known as isoprenoids, are a large group of chemicals derived from five-carbon isoprene units with diverse applications in pharmaceuticals, flavors, colorants, and even liquid fuel alternatives (Vickers et al., 2017; Zhou, 2018). As naturally produced organic chemicals, terpenoids are conventionally extracted from natural sources such as plants, but the long cultivation time and inefficiency in extraction limit the sufficient supply of terpenoids to meet the increasing demand (Chang and Keasling, 2006; Wang et al., 2018). Thus, microbial terpenoid production has gained much attention as an alternative way to produce terpenoids. To achieve high productivity and yield in microbial hosts, strong metabolic flux through the mevalonate (MVA) pathway or methylerythritol 4-phosphate (MEP) pathway are required to sufficiently supply isopentenyl pyrophosphate (Pompon et al., 1996) and thus geranyl pyrophosphate (GPP), the main precursor of terpenoids (Zhang and Hong, 2020). In addition, functional expression of heterologous enzymes adapted from a source organism plays a critical role in achieving high titer production of terpenoids. Here, we discuss the engineering efforts put into *S. cerevisiae* and *Y. lipolytica* for the production of a few representative compounds in each class of terpenoids and compare their performance to that of *E. coli*, a model bacterial cell factory.

#### Isoprene

**FIGURE 2:**
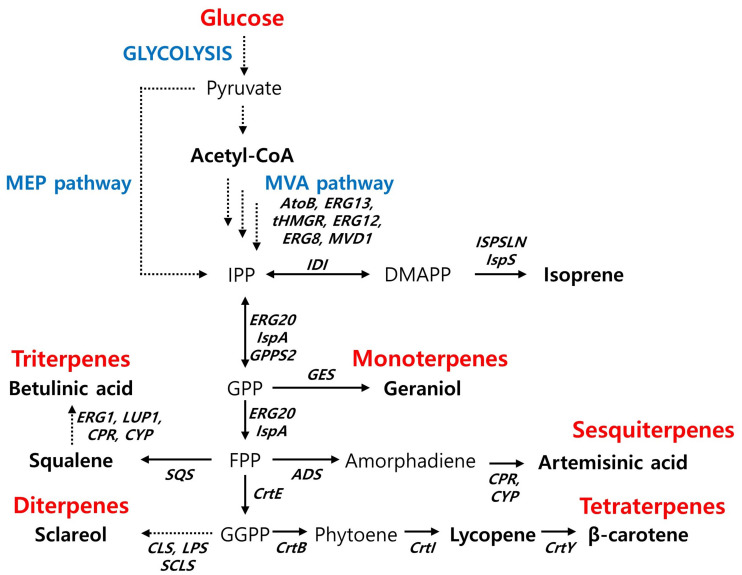
Biosynthetic pathway for terpenoids production and main engineering targets. AtoB, acetyl-CoA acetyltransferase; ERG13, hydroxymethylglutaryl-CoA synthase; tHMGR, truncated HMG-CoA reductase; ERG12, mevalonate kinase; ERG8, phosphomevalonate kinase; MVD1, diphosphomevalonate decarboxylase; IDI, isopentenl-diphosphate δ-isomerase; IspS, isoprene synthase; ISPSLN, mutant isoprene synthase; GPPS2, geranyl diphosphate synthase; ERG20 (IspA), farnesyl pyrophosphate synthethase; SQS, squalene synthase; ADS, amorpha-4,11-diene synthase; CPR, cytochrome P450 reductase; CYP, cytochrome P450 monooxygenase; ERG1, squalene monooxygenase; LUP1, lupeol synthase; CrtE, geranylgeranyl diphosphate synthase; CLS, 8-hydroxy copalyl diphosphate synthase; LPS, LDPP synthase; SCLS, sclareol synthase; CrtB, phytoene synthase; CrtY, lycopene beta-cyclase.

Isoprene (2-methyl-1,3-butadiene) is a colorless and flammable compound, and the main monomer of natural rubber. In *S. cerevisiae*, high-titer isoprene production (11.9 g/L) has been reported through protein engineering and enhanced precursor supply (Yao et al., 2018). Specifically, the authors constructed an isoprene production pathway by introducing a mutant isoprene synthase (ISPSLN), created by saturation mutagenesis, and compartmentalized a mitochondrial MVA pathway. In addition, improving the precursor supply through co-overexpression of diphosphomevalonate decarboxylase (MVD) and isopentenyl-diphosphate δ-isomerase, encoded by *MVD1* and *IDI1*, respectively, and redirection of the metabolic flux toward dimethylallyl pyrophosphate (DMAPP) contributed to improved metabolic flux through an isoprene synthetic pathway (Figure 2). There are no reports on the production of isoprene in *Y. lipolytica*, possibly due to its greater potential for the production of C30 or higher terpenoids (Larroude et al., 2018; Zhang et al., 2019). The isoprene titer reported by a bacterial counterpart of *E. coli* was 60 g/L. The engineered *E. coli* strongly expressed the heterologous MVA pathway and isoprene synthase (IspS) from *Populus alba* along with an enhanced pentose phosphate pathway (Figure 2). The isoprene productivity was 2 g/L/h with a yield of 850 mg/g DCW (Whited et al., 2010).

#### Geraniol (Monoterpene)

Geraniol (3,7-dimethylocta-trans-2,6-dien-1-ol) is one of the most widely used monoterpenes as a food additive, cosmetic ingredient, pesticide, and gasoline alternative. The highest titer of geraniol in *S. cerevisiae* has been reported to be 1.68 g/L through overexpression of the genes involved in the MVA pathway, tHMGR and IDI1, and the use of truncated geraniol synthase (GES) (Jiang et al., 2017; Figure 2). Of the nine candidates for heterologous GES, the GES from *Catharanthus roseus* (CrGES) showed the highest efficiency in geraniol production, which was further improved by truncation of a plastid targeting signal sequence required in its source organism. In addition, the researchers fused the ERG20ww (F96W/N126W), the modified FPPS solely generating GPP (Fischer et al., 2011; Ignea et al., 2014), into CrGES to enhance GPP accessibility (Figure 2). There are limited reports on monoterpene production by *Y. lipolytica* with a titer one order of magnitude lower than that by *S. cerevisiae*. Limonene and linalool have been produced at a titer of 165.3 mg/L (Cheng et al., 2019) and 6.96 mg/L (Cao et al., 2017), respectively, using minimally engineered *Y. lipolytica* strains. Geraniol production by *Y. lipolytica* has never been reported. Even with a distinctive cellular network and the lack of a native MVA pathway, the geraniol production efficiency of *E. coli* (Liu W. et al., 2016) has been shown to be similar to that of *S. cerevisiae* (2.0 vs. 1.68 g/L).

#### Artemisinic Acid (Sesquiterpene)

Artemisinic acid, a sesquiterpene with three isoprene units, is the precursor of artemisinin, a potent antimalarial drug (Figure 2). Based on the success by Ro et al. (2006), the highest titer of artemisinic acid (25 g/L) in *S. cerevisiae* has been reported through the construction of a functional biosynthetic route based on a plant-derived dehydrogenase and cytochrome P450 (Paddon et al., 2013). Possibly due to the already proven success in *S. cerevisiae*, no efforts have been devoted to the development of artemisinic acid-producing *Y. lipolytica*. Prior to the development of artemisinic acid-producing strain of *S. cerevisiae*, *E. coli* had been engineered through similar approaches involving cytochrome P450, but the titer was limited to only 105 mg/L due to the poor expression of cytochrome P450 in a prokaryotic cell factory of *E. coli* (Chang et al., 2007).

#### Sclareol (Diterpene)

Sclareol is a diterpene alcohol used as a cosmetic precursor in the fragrance industry. In *S. cerevisiae*, 750 mg/L of sclareol has been produced through protein engineering and deletion screening approaches (Trikka et al., 2015). To support sufficient metabolic fluxes in a sclareol synthesis pathway, a fusion protein of a modified ERG20 (F96C) and 8-hydroxycopalyl diphosphate synthase (CLS) from *Cistus creticus*, which catalyze the production of geranylgeranyl pyrophosphate (GGPP) and 8-hydroxycopalyl diphosphate (8-OH-CPP), respectively, were overexpressed in the strain harboring a sclareol synthase (SCLS), a class I diterpene synthase from *Salvia sclarea*. The engineered strain was further modified by disrupting the function of the genes selected from a heterozygous deletion screening experiments; this resulted in a 40-fold increase in the sclareol titer (750 mg/L). To our knowledge, the production of sclareol in *Y. lipolytica* has not been previously reported. In *E. coli*, a high titer (1.5 g/L) of sclareol was reportedly produced by simply introducing a heterologous pathway that converts GGPP into sclareol from *S. sclarea* (Schalk et al., 2012).

#### Betulinic Acid (Triterpene)

Betulinic acid is a pentacyclic triterpene and is used as an antitumor, antiviral, and anti-inflammatory agent (An et al., 2020). The highest titer of betulinic acid production obtained using *S. cerevisiae* was reported to be 182 mg/L by combining metabolic and process engineering (Czarnotta et al., 2017). The engineered strains used in the study overexpressed heterologous lupeol synthase (LUP1) and P450 reductase (CPR) from *Arabidopsis thaliana*, and P450 monooxygenase (CYP) from *Catharanthus roseus* along with homologous squalene monooxygenase (ERG1) and tHMGR. To increase the production titer of betulinic acid, these authors performed fed-batch fermentation with excess ethanol, which supports the increased supply of acetyl-CoA and NADPH for triterpene production. When combined with nitrogen-limitation conditions, metabolic flux could be directed toward betulinic acid synthesis and thereby increased the titer and yields of betulinic acid. In *Y. lipolytica*, betulinic acid titer of 51.87 mg/L has been reported during flask fermentation by adapting multimodular engineering strategy (Jin et al., 2019). To maximize betulinic acid production, they divided the betulinic acid synthesis pathway into four separate modules: the CYP/CPR, mevalonate, acetyl-CoA generation, and redox cofactor supply modules. The best combinations of heterologous CYP/CPR from five different sources, namely CYP from *Betula platyphylla* and CPR from *Medicago truncatula* were overexpressed along with ERG1, ylHMG1, and squalene synthase (ERG9); this resulted in the production of betulinic acid with a titer of 51.87 mg/L. Given that this titer was obtained from flask fermentation, the production performance of *Y. lipolytica* could be further improved when combined with process engineering approaches applied to *S. cerevisiae*. There are limited reports on the betulinic acid production in *E. coli* possibly due to the antibacterial properties of betulinic acid (Oloyede et al., 2017).

#### β-Carotene (Tetraterpene)

β-carotene is an orange-colored tetraterpene pigment composed of eight isoprene units, and is found in plants, photosynthetic bacteria, and algae. It exhibits antioxidant properties and serves as a precursor of vitamin A (Arendt et al., 2016). Recent success in engineering *S. cerevisiae* has improved β-carotene titer up to 750 mg/L (López et al., 2019) through the combined efforts of adaptive laboratory evolution and metabolic engineering; the strain overexpressing the carotenoid biosynthetic genes *crtYB*, *crtI*, and *crtE* from *Xanthophyllomyces dendrorhous* with *tHMGR were evolved for improved*β-carotene production. Later, high-titer production of lycopene, a precursor of β-carotene with its own utility, was reported to be 3.28 g/L (Shi et al., 2019) by expressing the heterologous genes involved in the lycopene biosynthesis pathway (*crtE*, *crtB*, *crtI* sourced from *Pantoea ananatis*, *Pantoea agglomerans*, and *Blakeslea trispora*, respectively) under the control of various promoters with optimized gene expression levels (Figure 2). Given that *crtB* and *crtI* are shared in the β-carotene synthesis pathway, the production titer of β-carotene could be further improved by introducing a proper gene at the last step of the β-carotene synthesis pathway. *Y. lipolytica* has shown superior potential for the production of β-carotene, with the highest titer of 6.5 g/L (Larroude et al., 2018). The high metabolic flux through acetyl-CoA and its lipophilic depository of liposomes provided a favorable cellular system for the high production of β-carotene. Introduction of a heterologous β-carotene synthesis pathway encoded by *carRP* and *carB* from *Mucor circinelloides* with overexpression of a native MVA pathway (ylHMG1) turned a previously engineered lipid over-producing strain of *Y. lipolytica* into a superior β-carotene-producing strain. A comparable β-carotene titer has been achieved by *E. coli* (Yang and Guo, 2014). The co-expression of an optimized MEP and a hybrid MVA pathway to maximize the supply of IPP and GPP resulted in a β-carotene titer of 3.2 g/L in the strain heterologously expressing the genes involved in the β-carotene synthesis pathway from *Erwinia herbicola* and GPPS2 from *Abies grandis*.

### Polyketides

Polyketides are a diverse group of secondary metabolites produced by bacteria (type I, II, III), fungi (type I, II), and plants (type III). Polyketides exhibit various bioactive properties such as anticancer, antifungal, and antiviral, and serve as important resources for pharmaceutical development (Jakočiūnas et al., 2020). Within the type III polyketides class, triacetic acid lactone, ρ-coumaric acid, naringenin, and resveratrol have been produced at high titers by fungal as well as bacterial hosts (López et al., 2019).

#### Triacetic Acid Lactone

**FIGURE 3:**
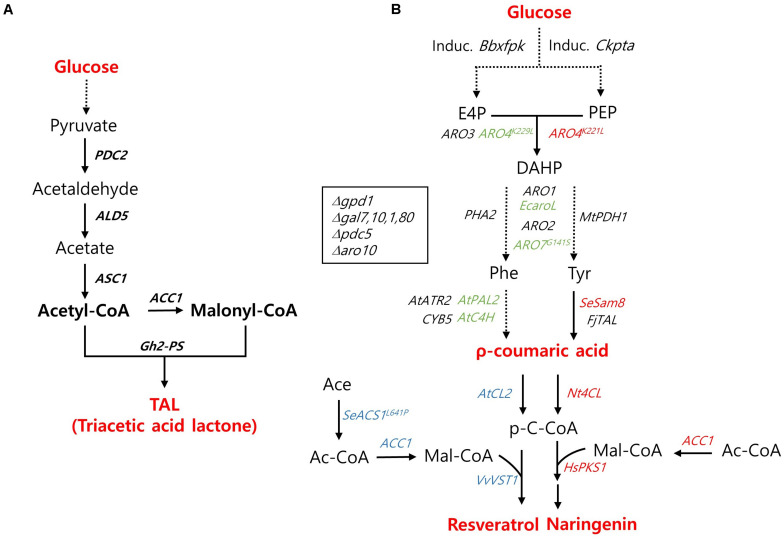
Biosynthetic pathway for polyketides production and main engineering targets. **(A)** A pathway for triacetic acid lactone production. **(B)** A pathway for the production of ρ-coumaric acid, resveratrol, and naringenin. The genes used specifically for the production ρ-coumaric acid, resveratrol, and naringenin are indicated with green, blue, and red colors, respectively. PDC, pyruvate decarboxylase; ALD5, aldehyde dehydrogenase 5; ACS1, acetyl-CoA synthase 1; ACC1, acetyl-CoA carboxylase; Gh2-PS, *Gerbera hybrida* 2-pyrone synthase; Induc. Bbxfpk, Induc. Ckpta, The inducible phosphoketolase (PHK) pathway including a *Bifidobacterium breve* phosphoketolase (Bbxfpk) and a *Clostridium kluyveri* phosphotransacetylase (Ckpta); ARO3, DAHP synthase; ARO4^*K*229L^, ARO4^*K*221L^, L-tyrosine-feedback-insensitive DAHP synthase; ARO1, pentafunctional arom protein; EcaroL, *Escherichia coli* shikimate kinase; ARO2, chorismate synthase; ARO7^*G*141S^, L-tyrosine-feedback-insensitive chorismate mutase; PHA2, prephenate dehydratase; MtPDH1, *Medicago truncatula* prephenate dehydrogenase; *At*C4H, *Arabidopsis thaliana* cinnamic acid hydroxylase; *At*PAL2, *A. thaliana* phenylalanine ammonia lyase; *At*ATR2, *A. thaliana* cytochrome P450 reductase; CYB5, Cytochrome b5; *Fj*TAL, *Flavobacterium johnsoniae* tyrosine ammonia lyase; SeSam8, *Saccharothrix espanaensis* tyrosine amino lyase; AtCL2, Nt4CL, *Arabidopsis thaliana*, *Nicotiana tabacum* 4-coumaroyl-CoA ligase; VvVST1, *Vitis vinifera* resveratrol synthase; SeACS1^*L*641P^, *Salmonella enteric* acetyl-CoA synthetase; HsPK1, *Huperiza serrata* chalcone synthase; gpd1, Glycerol-1-phosphatase; gal7, galactose-1-phosphate uridyl transferase; gal10, UDP-glucose-4-epimerase; gal1, Galactokinase; gal80, transcriptional regulator; aro10, phenylpyruvate decarboxylase.

Triacetic acid lactone (López et al., 2019), naturally produced by the plant *Gerbera hybrid*, is a simple polyketide used as a chemical building block for a range of value-added products such as sorbic acid, hexanoic acid, and acetylacetone (Chia et al., 2012). TAL is generated from two common metabolic precursors, acetyl-CoA and malonyl-CoA (Figure 3A). Recently, Markham et al. (2018) demonstrated TAL production in an engineered *Y. lipolytica* strain reaching a titer of 35.9 g/L, the highest amount obtained by recombinant strains during bioreactor operation (Table 1). Overexpression of *ACC1*, which encodes the enzyme that converts acetyl-CoA to malonyl-CoA, combined with overexpression of the pyruvate bypass pathway genes, *PDC2*, *ALD5*, and *ACS1*, and the β-oxidation pathway genes reinforced the already sufficient acetyl-CoA pool in *Y. lipolytica* leading to a high-titer TAL production (Figure 3A). In *S. cerevisiae*, the central carbon pathway is regulated toward TAL biosynthesis by blocking competing pathways and a protease enzyme activity resulting in 2.2 g/L of TAL production under bioreactor conditions (Cardenas and Da Silva, 2014). Moreover, TAL production in an engineered industrial strain of *S. cerevisiae* reached a titer of 5.2 g/L by employing fed-batch cultivation in a bioreactor with ethanol feeding (Saunders et al., 2015). In *E. coli*, a similar titer of TAL (2.1 g/L) was reported from the shake-flask culture of an engineered strain expressing a designed TAL reporter (Tang et al., 2013).

**TABLE 1:** Comparison of the production titer of non-native chemicals by engineered *S. cerevisiae*, *Y. lipolytica* and *E. coli*.

Product		Strain	Performance			Fermentation			References
Class	Name		Titer (g/L)	Yields (mg/g)	Productivity (mg/L/h)	Type	Mode	Main substrate	
Terpenoids	Isoprene	*S. cerevisiae*	11.90	N/A^*a*^	71*	B	Fed-batch	Glucose	Yao et al., 2018
		*E. coli*	60.00	110	2,000	B	Fed-batch	Glucose	Whited et al., 2010
	Geraniol	*S. cerevisiae*	1.68	N/A	14*	B	Fed-batch	Glucose	Jiang et al., 2017
		*E. coli*	2.00	N/A	29*	B	Fed-batch	Glucose	Liu W. et al., 2016
	Artemisinic acid	*S. cerevisiae*	25.00	N/A	180*	B	Fed-batch	Glucose	Paddon et al., 2013
		*E. coli*	0.105	N/A	N/A	F	Batch	Glycerol	Chang et al., 2007
	Betulinic acid	*S. cerevisiae*	0.182	N/A	N/A	B	Fed-batch	Glucose, ethanol	Czarnotta et al., 2017
		*Y. lipolytica*	0.052	N/A	N/A	F	Batch	Glucose	Jin et al., 2019
	Sclareol	*S. cerevisiae*	0.75	N/A	N/A	F	Batch	Glucose	Trikka et al., 2015
		*E. coli*	1.5	N/A	N/A	B	Fed-batch	Glycerol	Schalk et al., 2012
	β-carotene	*S. cerevisiae*	0.75	N/A	9.38*	B	Fed-batch	Glucose	López et al., 2019
		*Y. lipolytica*	6.50	N/A	53.28*	B	Fed-batch	Glucose	Larroude et al., 2018
		*E. coli*	3.20	N/A	61.54*	B	Fed-batch	Glycerol	Yang and Guo, 2014
	Lycopene	*S. cerevisiae*	3.28	N/A	25*	B	Fed-batch	Glucose	Shi et al., 2019
		*Y. lipolytica*	0.745	N/A	7.76*	B	Fed-batch	Glucose	Zhang et al., 2019
		*E. coli*	3.52	N/A	35.2*	B	Fed-batch	Glycerol	Sun et al., 2014
Polyketides	Triacetic	*S. cerevisiae*	2.20	130	N/A	B	Fed-batch	Glucose	Cardenas and Da Silva, 2014
	acid lactone		5.20	N/A	N/A	B	Fed-batch	Glucose, ethanol	Saunders et al., 2015
		*Y. lipolytica*	35.90	164	210	B	Batch	Glucose	Markham et al., 2018
		*E. coli*	2.10	N/A	N/A	F	Batch	Glycerol	Tang et al., 2013
			1.87	N/A	81.23*	F	Batch	Glycerol	Li et al., 2018
	ρ-coumaric acid	*S. cerevisiae*	12.50	139.6	130.1	B	Fed-batch	Glucose	Liu et al., 2019
		*E. coli*	0.974	N/A	27.06*	F	Batch	Glucose	Kang et al., 2012
		*E. coli*	0.24	N/A	5*	F	Batch	Glucose	Wu et al., 2017
	Naringenin	*S. cerevisiae*	0.22	N/A	11	F	Batch	Glucose	Lyu et al., 2019
		*Y. lipolytica*	0.898	N/A	N/A	B	Fed-batch	Glucose	Palmer et al., 2020
		*E. coli*	0.42	N/A	8.78*	F	Fed-batch	Glucose, tyrosine	Wu et al., 2015
	Resveratrol	*S. cerevisiae*	0.81	N/A	7.38*	B	Fed-batch	Glucose	Li et al., 2016
		*Y. lipolytica*	0.049	2.44	N/A	F	Batch	Glucose	Palmer et al., 2020
		*E. coli*	0.3045	75	6.34*	F	Batch	Glucose	Wu et al., 2017
FA-derived	Medium-chain	*S. cerevisiae*	2.82	N/A	29.3	B	Fed-batch	Glucose	Zhu et al., 2020
chemicals		*Y. lipolytica*	N/A	N/A	N/A	F	Batch	Glucose	Rigouin et al., 2018
	fatty acids	*E. coli*	1.36	N/A	N/A	F	Batch	Glycerol	Sherkhanov et al., 2014
	Odd-chain	*S. cerevisiae*	0.002	N/A	N/A	F	Batch	Glucose	Jin et al., 2016
	fatty acids	*Y. lipolytica*	0.36	N/A	N/A	F	Batch	Glucose	Park et al., 2020
			0.57	N/A	N/A	F	Fed-batch	Glucose, propionate	Park et al., 2018
		*E. coli*	0.297	N/A	N/A	F	Batch	Glucose, propionate	Wu and San, 2014
	Fatty alcohols	*S. cerevisiae*	6.00	58	N/A	B	Fed-batch	Glucose	d’Espaux et al., 2017
		*Y. lipolytica*	5.8	36	N/A	B	Fed-batch	Glucose	Cordova et al., 2020
		*E. coli*	6.33	N/A	N/A	B	Fed-batch	Glycerol	Liu Y. et al., 2016
Others	PHB	*S. cerevisiae*	0.73	13.8	4.3*	F	Batch	Xylose	Portugal-Nunes et al., 2017
		*Y. lipolytica*	7.35	N/A	N/A	B	Fed-batch	Glucose	Li et al., 2017
		*E. coli*	4.13*	360	N/A	F	Batch	Glucose	Wang J. et al., 2020
	Muconic acid	*S. cerevisiae*	20.8	66.2	139	B	Fed-batch	Glucose	Wang G. et al., 2020
		*E. coli*	4.09	310	56.8	F	Batch	Glucose, xylose	Fujiwara et al., 2020

^a^Not available. *Calculated data. B, bioreactor; F, flask; P, 24 deep-well plate.

#### ρ-Coumaric Acid

ρ-Coumaric acid is an aromatic amino acid (AAA) that serves as a starting material for numerous high-value biochemicals such as flavors, fragrances, nutraceuticals, and pharmaceuticals. Recently, a recombinant diploid QL60 strain of *S. cerevisiae* produced 12.5 g/L of ρ-coumaric acid with a productivity of 0.13 g/L/h and a yield of 0.14 g/g sugar under glucose-limited fed-batch fermentation conditions (Liu et al., 2019). This demonstrated superior performance of *S. cerevisiae* in the production of ρ-coumaric acid over an *E. coli* strain, in which 168–974 mg/L was produced during flask culture (Kang et al., 2012; Morelli et al., 2017). This remarkable performance of ρ-coumaric acid biosynthesis in *S. cerevisiae* was achieved by intensive engineering; systematic engineering of the AAA pathway through debottlenecking a shikimate pathway, enhancing cytochrome P450 activity by overexpression of *AtCYB5* and *AtATR2*; and diverting carbon flux from glycolysis to erythrose 4-phosphate (E4P) by introducing a heterologous phosphoketolase (PHK)-based pathway. Optimization of carbon flux through interconnecting points between glycolysis and the AAA pathway was also critical for high-titer ρ-coumaric acid production (Figure 3B).

#### Naringenin

Naringenin is a key bioactive polyketide derived from ρ-coumaric acid, from which numerous flavonoids can be synthesized. Palmer et al. (2020) demonstrated *de novo* production of naringenin from *Y. lipolytica*, with the highest titer among those using *S. cerevisiae* and *E. coli*. In this study, they introduced the tyrosine-based ρ-coumaroyl-CoA- naringenin pathway and enhanced the metabolic flux from acetyl-CoA to malonyl-CoA through the overexpression of *ACC1* and *PEX10* (Figure 3B). The effectiveness of the upregulation of the peroxisomal matrix protein encoded by *PEX10*, previously reported to strongly improve the production of TAL, increased acetyl-CoA pool via refluxing the β-oxidation pathway. Besides, the introduction of mutant and/or heterologous enzymes, ARO4^*K*221L^ and a tyrosine amino lyase encoded by *SeSam8* from *Saccharothrix espanaensis*, resolved the rate-limiting steps contributing to the higher titer (Figure 3B). The final strain of engineered *Y. lipolytica* produced 124.1 mg/L of naringenin in flask culture, which was further increased to 898 mg/L during bioreactor operation. Lyu et al. (2019) applied multiple approaches for enhancing the metabolic flux toward naringenin via a tyrosine-based pathway. More interestingly, they investigated the beneficial effects of the co-culture system, in which the whole pathway was divided between two strains to relieve the heavy burden of complicated biosynthesis pathways and surfactant supplementation (Tween 80) on the improved naringenin production in *S. cerevisiae* (Lyu et al., 2019). In a flask culture, the naringenin titer of *S. cerevisiae* was higher than that of *Y. lipolytica* (220 vs. 124.1 mg/L), implying the potential of *S. cerevisiae* as a production host of naringenin as demonstrated in resveratrol production (discussed in the next paragraph). In *E. coli*, 421.6 mg/L of naringenin was produced in flask culture condition (Wu et al., 2015).

#### Resveratrol

Resveratrol shares its precursor with naringenin; ρ-coumaroyl-CoA is converted either to resveratrol or naringenin by stilbene synthase (STS) or the serial reaction of chalcone synthase (CHS) and chalcone isomerase (CHI), respectively. With the introduction of a phenylalanine-based ρ-coumaroyl-CoA—route, *S. cerevisiae* demonstrated higher resveratrol productivity over *Y. lipolytica* or *E. coli*. Li et al. adapted the engineering strategy used for ρ-coumaric acid production; overexpression of AtATR2, ARO1/2, and ARO3 encoded by *AtPAL2*, *EcaroL*/*ARO7*^*G*141*S*^, and *RO4*^*K*229*L*^, and enhanced availability of malonyl-CoA by overexpressing *SeACS*^*L*641*P*^, *ACC1*^*S*659*A, S*1157*A*^ encoding acetyl-CoA synthetase and acetyl-CoA carboxylase (Li et al., 2016) along with *ARO10* deletion (Figure 3B; Li et al., 2016). As mentioned above, *S. cerevisiae* efficiently produced 812 mg/L of resveratrol during fed-batch fermentation in a bioreactor, which was similar to the naringenin production of *Y. lipolytica* under bioreactor conditions (Table 1). Under shake-flask conditions, *Y. lipolytica* produced 48.7 mg/L of resveratrol (Palmer et al., 2020). In *E. coli*, 304.5 mg/L of resveratrol was produced in flask culture conditions (Wu et al., 2017; Table 1). Despite the lack of post-transcriptional modification, *E. coli* has shown a higher-titer production of plant-based polyketides. The main engineering strategies employed in *E. coli* mainly focused on increasing the malonyl-CoA pool and balancing the synthetic pathway via gene expression regulation. Recent reports on the newly developed CRISPRi system, which controls gene expression through repression rather than conventional gene knock-out, applied in *E. coli* offered finely tuned metabolic flux effectively delivered through the newly introduced synthetic pathway (Wu et al., 2015, 2017).

### Fatty Acid-Derived Chemicals

Fatty acids are essential compounds for sustaining cell membranes. With the help of synthetic biology, fatty acids are transformed into non-native chemicals with a wide range of applications. As fatty alcohols and fatty esters have been frequently discussed in previous reviews (Hu et al., 2019; Munkajohnpong et al., 2020), we focused on medium-chain and odd-chain fatty acids for which yeast engineering efforts have made noticeable progress in recent years, due to their importance as platform chemicals for the replacement of petroleum in the chemical industry.

#### Medium-Chain Fatty Acids (MCFAs)

**FIGURE 4:**
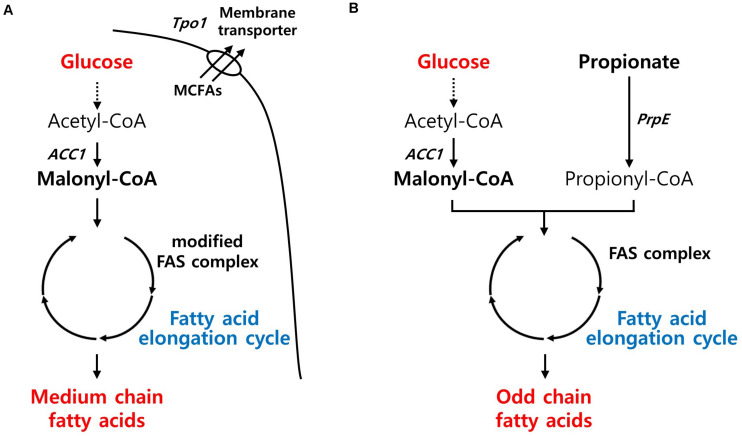
Biosynthetic pathway for fatty-acids derived chemicals production and main engineering targets. **(A)** A pathway for MCFAs production. **(B)** A pathway for OCFAs production. ACC1, acetyl-CoA carboxylase; Tpo1, polyamine transporter; PrpE, propionate-CoA ligase; FAS, fatty acid synthase.

MCFAs have gained attention as jet fuel replacements, platform chemicals, and ingredients for plastics and cosmetics. Recently, an MCFA-producing *S. cerevisiae* strain was developed by adapting a type I bacterial fatty acid synthesis pathway. Through the directed evolution of TPO1 transporter and laboratory adaptive evolution, the engineered strain with improved MCFA tolerances produced almost 1 g/L of MCFAs (Zhu et al., 2020; Figure 4A). In *Y. lipolytica*, the native FAS enzyme was simply modified to synthesize MCFAs based on its superior capacity for long-chain fatty acid (LCFA) production. Although the MCFA content (45%) in its total lipids was agreeable, the titer was insignificant, possibly because its FAS systems evolved toward a highly efficient LCFA production system (Rigouin et al., 2017). *E. coli* has also shown a similar range of MCFA titer (1.36 g/L) through metabolic engineering approaches along with enhancing the tolerance toward MCFAs (Sherkhanov et al., 2014).

#### Odd-Chain Fatty Acids (OCFAs)

In nature, most fatty acids derived from microorganisms contain even numbers of carbon, and rare microbes produce minute amounts of OCFAs with the exception of propionic acid. Recently, microbial production of OCFAs has also attracted considerable interest because of its potential benefits to human health, such as diabetes (Weitkunat et al., 2017) or adiposity (Aglago et al., 2017). Modifying the fatty acid synthesis pathway, in which acetyl-CoA and malonyl-CoA are used as starting materials, is not simple unless odd chain fatty acids such as propionic acid are fed as substrates. Recently, *Y. lipolytica* has shown promise as a cell factory for the production of OCFAs by harnessing the power of superior LCFA production capacity. When the metabolic flux toward LCFA production in an obese strain diverged toward OCFAs, *Y. lipolytica* produced 360 mg/L of OCFAs with a chain length of over C15. By supplying endogenous propionyl-CoA from oxaloacetate through a newly introduced synthetic pathway, the engineered strain with the genetic background for high lipid production was able to produce OCFAs without the supplementation of propionic acid (Park et al., 2020; Figure 4B). In *E. coli*, the overexpression of propionyl-CoA synthase from *Salmonella enterica* and acyl-ACP thioesterase from *Ricinus communis* and *Umberllularia california* enabled the production of 276 mg/L of OCFAs, with chain lengths of C11–C15, comparable to that in *Y. lipolytica*, in the presence of optimized propionate supplementation (Wu and San, 2014).

### Other Chemicals

#### Polyhydroxybutyrate (PHB)

**FIGURE 5:**
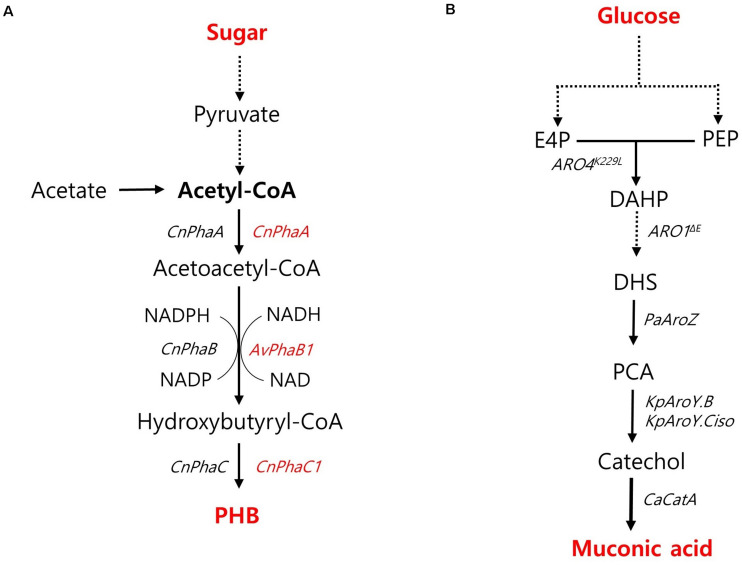
Biosynthetic pathway for other chemicals production and main engineering targets. **(A)** A pathway for PHB production. The genes specifically used for *S. cerevisiae* and *Y. lipolytica* are indicated with red and black colors, respectively. **(B)** A pathway for muconic acid production. *Cn*, *Cupriavidus necator*; *Av, Allochromatium vinosum; PhaA*, acetyl-CoA acetyl transferase; PhaB, acetoacetyl-CoA reductase; *PhaC*, polyhydroxyalkanoate synthase. ARO4^*K*229L^, L-tyrosine-feedback-insensitive DAHP synthase; *ARO1*^Δ*E*^, AROM protein without shikimate dehydrogenase domain; *PaAroZ*, *Podospora anserine* DHS dehydratase; *KpAroY.B*, *KpAroY.Ciso*, *Klebsiella pneumonia* PCA decarboxylase subunits; *CaCatA*, *Candida albicans* catechol 1,2-dioxygenase.

PHB has recently emerged as a promising candidate for the sustainable production of biodegradable thermoplastics. PHB is naturally accumulated as energy storage molecules under non-optimal conditions by several bacterial species such as *Cupriavidus necator* (previously known as *Ralstonia eutropha*) and *Methylobacterium*. However, these native producers still have drawbacks to be served as a production host for PHB commercialization due to their intracellular lysis of PHB, poor tolerance toward harsh environments, restriction of metabolic substrates, and unfamiliar genetic characterization (Madison and Huisman, 1999). Thus, the feasibility of PHB production in non-native hosts has been investigated. High-titer PHB production was achieved by an engineered oleaginous yeast *Y. lipolytica* harboring a heterologous PHB biosynthetic pathway based on *PhaA, PhaB*, and *PhaC* from a native producer of *C. necator* (Figure 5A; Li et al., 2017). To strengthen the availability of cytosolic acetyl-CoA, acetate, an essential precursor for PHB production, was fed as a sole carbon source, resulting in a PHB content of 10.2% with a high titer of 7.35 g/L during fed-batch fermentation in a bioreactor (Li et al., 2017). In *S. cerevisiae*, high PHB content was achieved during xylose fermentation (Portugal-Nunes et al., 2017). An engineered xylose-utilizing strain of *S. cerevisiae* with a heterologously expressed PHB pathway based on *PhaA* and *PhaC1* from *C. necator* and NADH-preferred *PhaB1* from *Allochromatium vinosum* (Figure 5A) produced 730 mg/L PHB with a content of 16.4% during xylose fermentation with additional nitrogen supply under an anaerobic shake-flask culture condition (Portugal-Nunes et al., 2017). PHB production in *E. coli* has been shown to be more promising than that in yeast strains. Wang et al. reported the role of truncated lipopolysaccharide (LPS) in *E. coli* in improving PHB production as a rebalanced carbon and nitrogen metabolism with increased precursors and energy levels. By introducing a PHB synthesis pathway based on PhaA, PhaB, and PhaC from *C. necator* (Figure 5A) as well as a truncated LPS, the engineered strain of *E. coli* produced PHB with a superior content and yield of 84.3% and 360 mg/g, respectively, under shake-flask culture conditions (Wang J. et al., 2020). In terms of PHB content and yield, the production capacity of the engineered *E. coli* was highly competitive with the natural PHB-producing strain of *C. necator*, which produced 232 g/L of PHB with a content of 80% and a yield of 380 mg/g during glucose fed-batch fermentation under phosphate limitation conditions in a bioreactor (Ryu et al., 1997).

#### Muconic Acid

Muconic acid is another platform chemical with the potential for production of bio-plastics, such as nylon-6,6, polyurethane and polyethylene terephthalate, as well as cosmetics and pharmaceuticals (Xie et al., 2014). Wang G. et al. (2020) developed high muconic acid-producing *S. cerevisiae* through biosensor-aided genome engineering. From a UV-mutagenesis library, they screened out a mutant strain harboring missense mutations in several native genes, such as *PWP2*, *EST2*, *ATG1*, *DIT1*, *CDC15*, *CTS2*, and *MNE1*, duplicated *aroZ*, and *catA*, which are involved in the introduced muconic acid pathway (Figure 5B). The overexpression of protocatechuic acid (PCA) decarboxylase and AROM protein without the shikimate dehydrogenase domain (Aro1p^Δ *E*^) led to the high production of muconic acid, resulting in a muconic acid titer of 20.8 g/L during pH-controlled fed-batch fermentation. In *E. coli*, co-fermentation of glucose and xylose resulted in 4.09 g/L of muconic acid in a shake-flask condition (Fujiwara et al., 2020; Table 1).

## Challenges and Opportunities in Non-Native Chemical Production

Based on success as a proven industrial cell factory for biofuel production, yeasts are transforming into a cell factory for the production of non-native chemicals. In addition to the benefit from their robustness, yeasts offer ease of reactor operation and cell separation as well as low energy consumption with lower fermentation temperature (Liu et al., 2019). By exploiting the advantages of eukaryotic systems, yeast demonstrated its potential for the biosynthesis of plant-derived metabolites by better supporting the functional expression of heterologous enzymes requiring post-translational modification or providing intracellular structures for proper enzyme activity (Siddiqui et al., 2012). For instance, membrane-bound cytochrome P450 oxidase, a crucial enzyme for the biosynthesis of plant metabolites, can be functionally expressed in yeast (Pompon et al., 1996). Due to these advantages, the two main yeast cell factories, namely *S. cerevisiae* and *Y. lipolytica*, could potentially be used for the production of plant-derived chemicals, especially terpenes with three or more isoprene units and polyketides. *S. cerevisiae* has been shown to produce higher titers of artemisinic and betulinic acids. Moreover, *Y. lipolytica* has shown comparable performance with *S. cerevisiae* for the production of β-carotene and lycopene (in terms of titer), even with relatively minimal engineering. The higher titers in terpene production by *S. cerevisiae* were often based on subcellular compartmentalization using mitochondria or peroxisomes, implying the critical role of proper intracellular structures. The potential of *Y. lipolytica* in triterpene production could be improved by adapting the engineering strategy of compartmentalization applied to *S. cerevisiae*. In the strain development for the biosynthesis of β-carotene and lycopene in *S. cerevisiae* and *Y. lipolytica*, balanced enzyme expression levels played a critical role in enhancing production titer, especially at the rate-limiting steps mediated by *crtI* and *crtE*, the two main engineering targets. Even with limited attempts, *Y. lipolytica* has shown greater titer in the production of β-carotene, naringenin, and TAL by taking advantage of the high accessibility of acetyl-CoA over *S. cerevisiae* and *E. coli*. As more engineering tools are developed, and thus more sophisticated tuning of gene expression would be possible, *Y. lipolytica* could be used as a promising industrial host for the production of tetraterpene such as β-carotene. Moreover, polycistronic gene expression systems, which allow equivalent expression and regulation of multiple genes by one promoter in bacterial hosts, have been recently investigated and successfully developed for fungal hosts such as *S. cerevisiae* (Souza-Moreira et al., 2018). Therefore, the construction of a non-native chemical production pathway involving numerous heterologous genes would facilitate the development of yeast cell factories. Interestingly, unconventional carbon sources other than glucose, such as xylose, acetate, and propionic acid, have been shown to better support metabolic flux through the biosynthesis pathway for non-native chemicals (Wu and San, 2014; Li et al., 2017; Portugal-Nunes et al., 2017; Wu et al., 2019). Thus, non-native chemical production by the engineered yeast strains with non-native carbon metabolism could offer better promises for industrial-scale production of non-native chemicals.

## Conclusion

In this review, we discussed the current status of non-native biochemical production by model and non-model yeasts of *S. cerevisiae* and *Y. lipolytica*, respectively. The comparison of the potential of these yeasts in the production of non-native chemicals presented can serve to identify the production host of choice. By taking advantage of their distinctive cellular metabolism and characteristics as eukaryotic systems, these yeasts offer great potential for the industrial production of various non-native chemicals. Recent technological developments, such as high-throughput screening methods, are accelerating the application of synthetic biology to develop and upgrade yeast cell factories in a more efficient manner. Through target-specific engineering strategies with an expanded carbon source portfolio and optimized fermentation conditions of a single/co-culture system, yeasts could be successfully transformed into favorable industrial cell factories for the production of a wide range of non-native chemicals.

## Author Contributions

JK and PH wrote and edited the manuscript. S-ML provided conception of this review and edited the manuscript. All authors contributed to the article and approved the submitted manuscript.

## Conflict of Interest

The authors declare that the research was conducted in the absence of any commercial or financial relationships that could be construed as a potential conflict of interest.
